# Supplementary material for: SIRT7 has a critical role in bone formation by regulating lysine acylation of SP7/Osterix
Source: Nat Commun. 2018 Jul 19;9:2833. doi: 10.1038/s41467-018-05187-4 (PMC6053369; doi:10.1038/s41467-018-05187-4)
Supplement: Supplementary file 1 — Supplementary Information [file 41467_2018_5187_MOESM1_ESM.pdf]

# SIRT7 has a critical role in bone formation by regulating lysine acylation of SP7/Osterix

**Fukuda, Yoshizawa, Karim *et al*.**

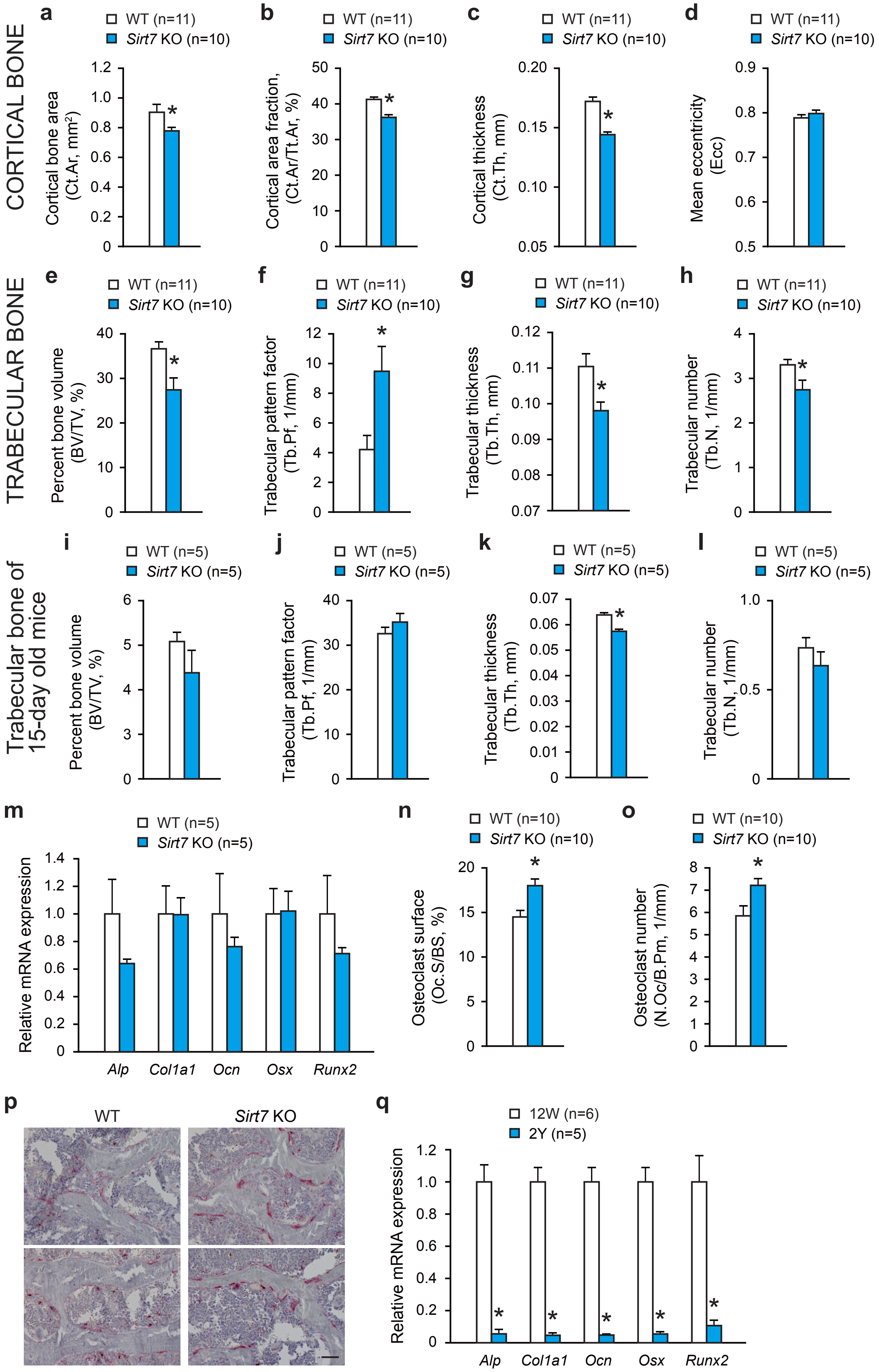

CORTICAL BONE
a
WT (n=11)
Sirt7 KO (n=10)
Cortical bone area (Ct.Ar, mm²)
b
Cortical area fraction, (Ct.Ar/Tt.Ar, %)
c
Cortical thickness (Ct.Th, mm)
d
Mean eccentricity (Ecc)
TRABECULAR BONE
e
Percent bone volume (BV/TV, %)
f
Trabecular pattern factor (Tb.Pf, 1/mm)
g
Trabecular thickness (Tb.Th, mm)
h
Trabecular number (Tb.N, 1/mm)
Trabecular bone of 15-day old mice
i
WT (n=5)
Sirt7 KO (n=5)
j
k
l
m
Relative mRNA expression
Alp
Col1a1
Ocn
Osx
Runx2
n
WT (n=10)
Sirt7 KO (n=10)
Osteoclast surface (Oc.S/BS, %)
o
Osteoclast number (N.Oc/B.Pm, 1/mm)
p
WT
Sirt7 KO
q
12W (n=6)
2Y (n=5)

**Supplementary Figure 1: Severe osteopenia in *Sirt7* KO mice.**
(**a**-**h**) μCT analysis of the distal femur in male *Sirt7* KO mice and WT mice aged 15-16 weeks. Ct.Ar (**a**), Ct.Ar/Tt.Ar (**b**), Ct.Th (**c**), Ecc (**d**), trabecular BV/TV (**e**), Tb.Th (**f**), Tb.N (**g**), and Tb.Pf (**h**).
(**i**-**l**) μCT analysis of the distal femur in female *Sirt7* KO mice and WT mice aged 15 days. trabecular BV/TV (**i**), Tb.Th (**j**), Tb.N (**k**), and Tb.Pf (**l**).
(**m**) Expression of osteoblastic marker genes analyzed by qRT-PCR in femurs harvested from female *Sirt7* KO mice and WT mice aged 15 days.
(**n**-**p**) Static and dynamic bone histomorphometric analyses of the lumbar spine (L4) in female *Sirt7* KO mice and WT mice aged 14-15 weeks. Oc.S/BS (**n**), N.Oc/B.Pm (**o**), and representative TRAP-stained images (**p**).
(**q**) Expressions of osteoblastic marker genes analyzed by qRT-PCR in femurs harvested from young (12 weeks old) and aged (2 years old) WT mice.
Data are shown as the mean± SEM. *$p < 0.05$. vs. WT. Scale bar, 100 μm.

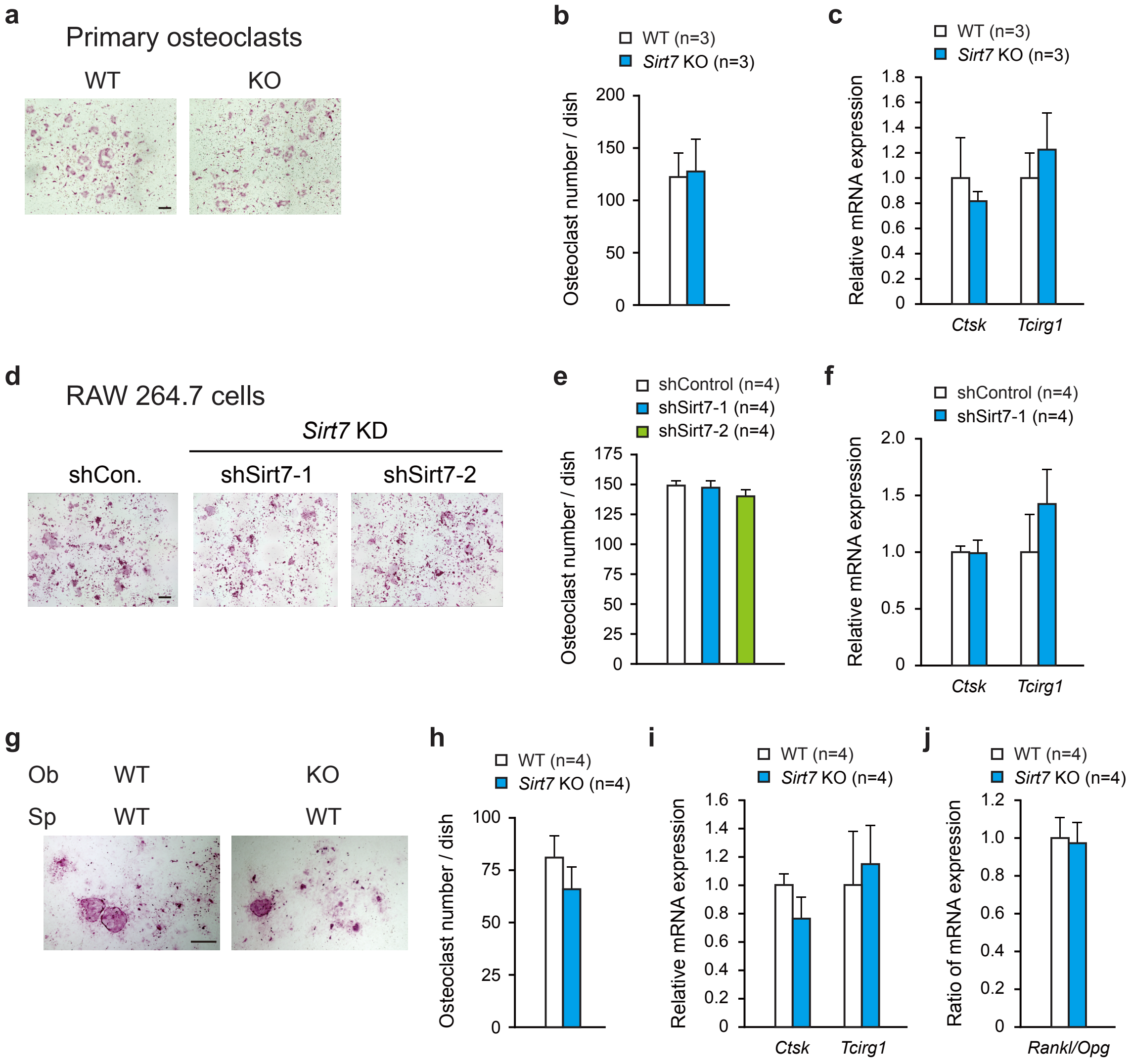


**Supplementary Figure 2: Osteoclastic and osteoblastic SIRT7 is not essential for osteoclast differentiation *in vitro*.**

(**a**-**c**) Osteoblast-free osteoclast differentiation assay. Monocytes/macrophages derived from the bone marrow of *Sirt7* KO mice and WT mice were cultured with RANKL for 5 days.

(**d**-**f**) Osteoblast-free osteoclast differentiation assay. *Sirt7* knockdown RAW264.7 cells and control cells were cultured with RANKL for 5 days.

(**g**-**j**) Osteoclast formation in co-culture. Calvarial osteoblasts (Ob) isolated from *Sirt7* KO mice and WT mice were cultured with WT splenocytes (Sp) and 1,25-dihydroxyvitamin $D_3$ for 15 days.

Representative TRAP-stained images (**a**,**d**,**g**), number of TRAP-positive multinucleated osteoclasts (**b**,**e**,**h**), expression of osteoclast marker genes analyzed by qRT-PCR (**c**,**f**,**i**), and *Rankl*/*Opg* mRNA expression ratio analyzed by qRT-PCR (**j**).

Data are shown as the mean ±SEM. *$p < 0.05$. vs. WT (**b**,**c**,**h**,**i**,**j**), or shControl (**e**,**f**). Scale bar, 300 μm.

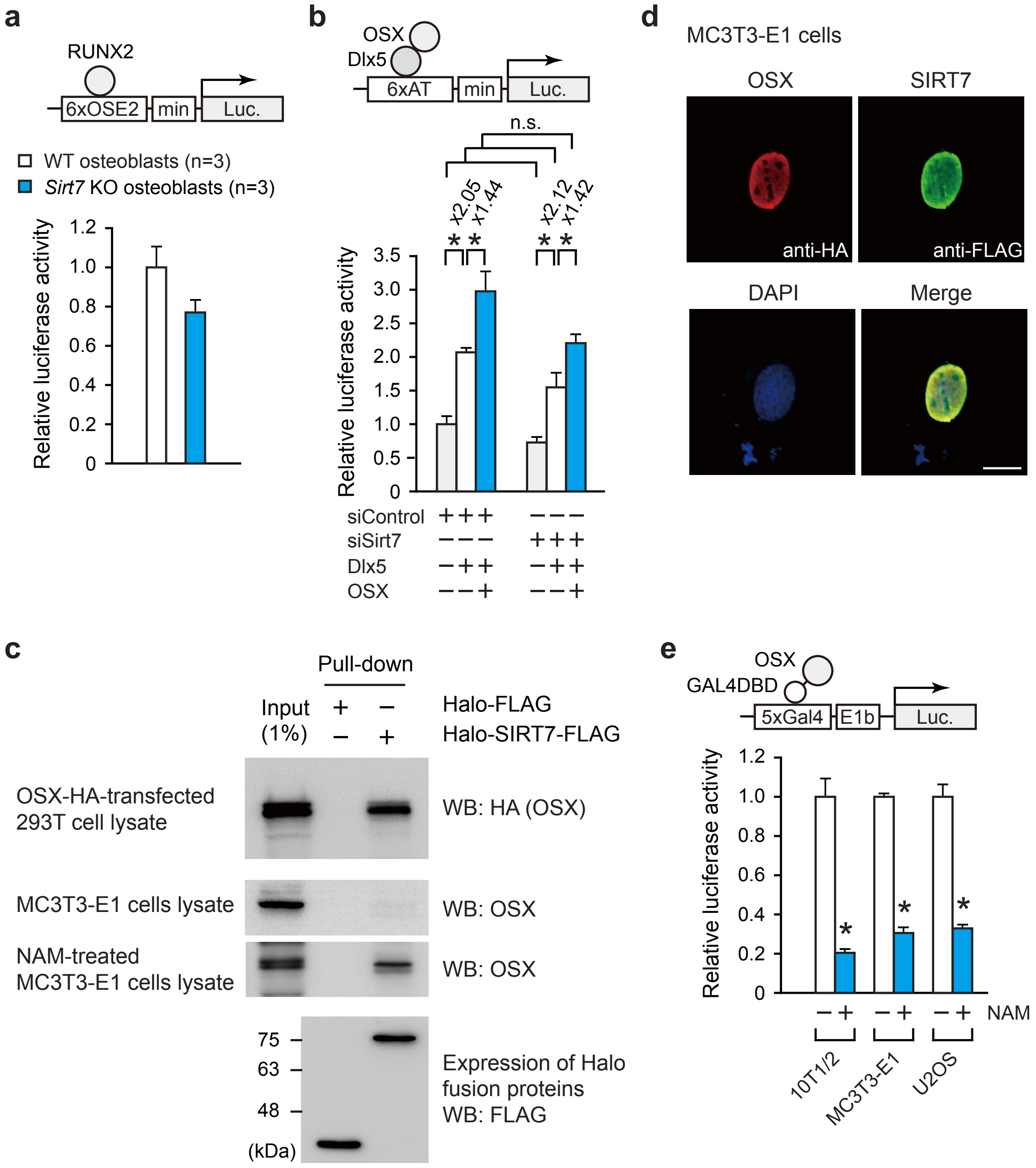


**Supplementary Figure 3: SIRT7 interacts with OSX and increases its transcriptional activity.**

(**a**) Transcriptional activity of RUNX2 in *Sirt7* KO and WT osteoblasts analyzed by the luciferase assay. Cells were transfected with the RUNX2 expression plasmid and the 6×OSE2-luciferase reporter plasmid. Luciferase activity was determined 24 hours after transfection.

(**b**) Transcriptional activity of Dlx5-OSX complex in MC3T3-E1 cells. After the indicated siRNA was introduced, MC3T3-E1 cells were cultured for 48 hours in differentiation medium supplemented with 10 ng/ml recombinant human BMP-2 (rhBMP-2, a gift from Osteopharma Inc., Osaka, Japan). Then cells were transfected with the indicated expression plasmid and the 6×AT-luciferase reporter plasmid, and the

reporter assay was performed after 24 hours (n=4 each).

(**c**) Halo-SIRT7-FLAG pull-down assay performed with lysates of OSX-HA transfected HEK293T cells and MC3T3-E1 cells incubated with/without 10 mM NAM. Overexpressed OSX-HA and endogenous OSX were detected by WB with an anti-HA antibody and anti-OSX antibody, respectively.

(**d**) Intracellular localization of OSX and SIRT7. MC3T3-E1 cells were co-transfected with the pcDNA3-3×HA-OSX and pcDNA3-FLAG-SIRT7 expression plasmids. Cells were stained with anti-HA antibody (Alexa Fluor 563; red) and anti-FLAG antibody (Alexa Fluor 488; green). DAPI (blue) was used for nuclear staining.

(**e**) Transcriptional activity of OSX in 10T1/2, MC3T3-E1, and U2OS cells. Cells were transfected with the GAL4DBD-OSX expression plasmid and the 5×GAL4-luciferase reporter plasmid, and were treated with 10 mM NAM 24 hours later. Luciferase activity was determined after 24 hours of NAM treatment. n=3 each.

WB=western blotting, IP=immunoprecipitation.

Data are shown as the mean ± SEM. *$p < 0.05$. vs. WT (**a**), or without NAM (**e**). Scale bar, 10 μm.

**a**

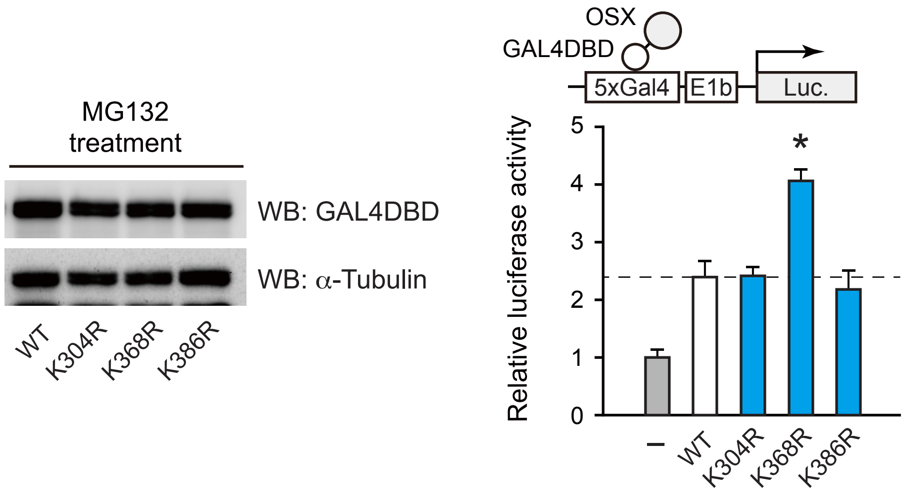


**b**

| | |
|---|---|
| Human (Homo sapiens) | T R S D H L S **K** H Q R T H |
| Mouse (Mus musculus) | T R S D H L S **K** H Q R T H |
| Bird (Cuculus canorus) | T R S D H L S **K** H Q K T H |
| Frog (Xenopus tropicalis) | T R S D H L S **K** H Q R S H |
| Zebrafifh (Danio rerio) | T R S D H L S **K** H Q K T H |

**Supplementary Figure 4: Lysine 368 is important for transactivation activity of mouse OSX.**

(**a**) Transactivation activity of OSX KR mutants in MC3T3-E1 cells treated with MG132. Cells were transfected with the GAL4DBD-OSX WT expression plasmid or the indicated GAL4DBD-OSX mutant expression plasmid, as well as the 5×GAL4-luciferase reporter plasmid. After 6 hours, the cells were treated with 10 μM MG132 for 24 hours, and luciferase activity was determined (right) (n=6 each). GAL4DBD-OSX protein was analyzed by WB under these conditions (left).

(**b**) Alignment of the C-terminal domains of OSX from different species. The lysine 368 of mouse OSX (magenta) is highly conserved in the indicated vertebrates.

WB=western blotting. Data are shown as the mean ± SEM. *p < 0.05 vs. GAL4DBD-WT OSX (**a**).

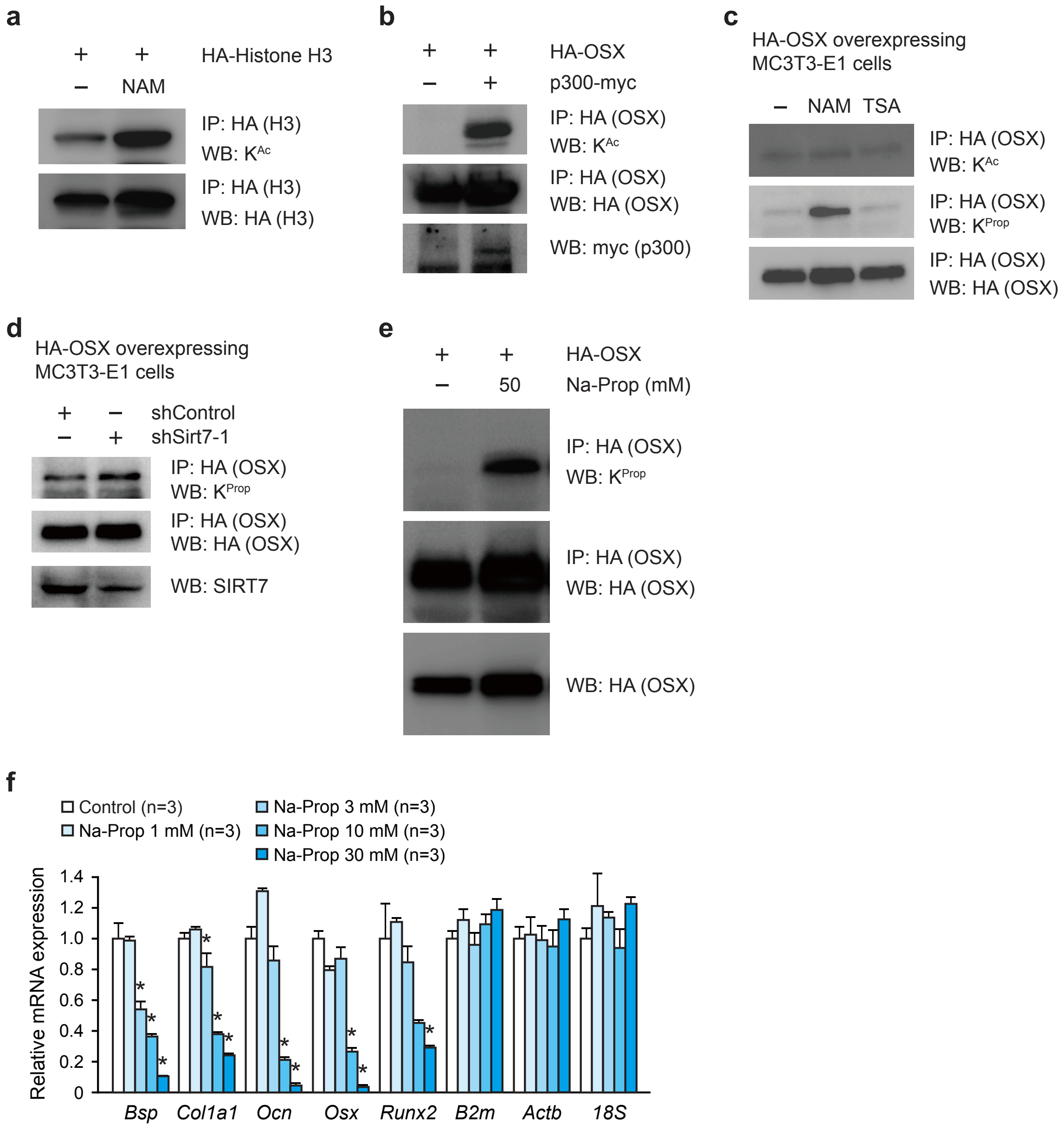


**Supplementary Figure 5: Lysine propionylation of OSX is regulated by SIRT7.**

(**a,b**) Detection of acetylated histone H3 and OSX. HEK293T cells were transfected with the HA-histone H3 expression plasmid and treated with 10 mM NAM for 24 hours (**a**). HEK293T cells were transfected with the 3×HA-OSX expression plasmid and the p300-myc or empty expression plasmid (**b**). After immunoprecipitation, acetylation of histone H3 and OSX were assessed by WB.

(**c**) Detection of acetylated or propionylated OSX. MC3T3-E1 cells stably overexpressing 3×HA-OSX were treated with 10 mM NAM for 24 hours or with 1 mM TSA for 6 hours. After immunoprecipitation, acetylated and propionylated OSX were detected by WB. A long exposure time was needed to detect acetylated OSX.

(**d**) Effect of *Sirt7* deficiency on propionylation of OSX. The indicated shRNA was introduced into 3×HA-OSX-overexpressing MC3T3-E1 cells. Propionylation of OSX

was assessed by immunoprecipitation and WB.

(**e**) Treatment with Na-Prop stimulates propionylation of OSX. MEF were transfected with the 3×HA-OSX expression plasmid and were treated with 50 mM Na-Prop for 16 hours. Then propionylation of OSX was assessed by immunoprecipitation and WB.

(**f**) Impact of propionylation on early differentiation of MC3T3-E1 cells. After the cells were cultured for 3 days in differentiation medium supplemented with various concentrations of Na-Prop, expression of osteoblast markers and non-osteoblast related genes was analyzed by qRT-PCR.

WB=western blotting, IP=immunoprecipitation.

Data are shown as the mean ± SEM. *$p < 0.05$. vs. Control (**f**)

**a**

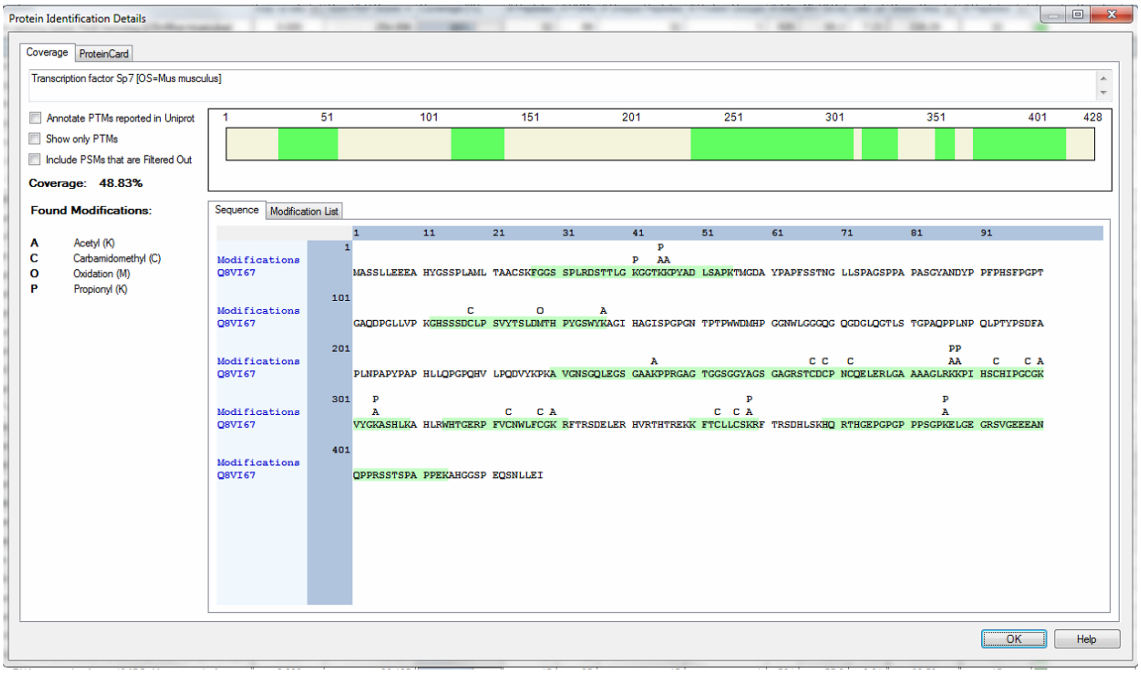


**b**

**K7Prop (OSX K41)**

D S T T L G k G G T K

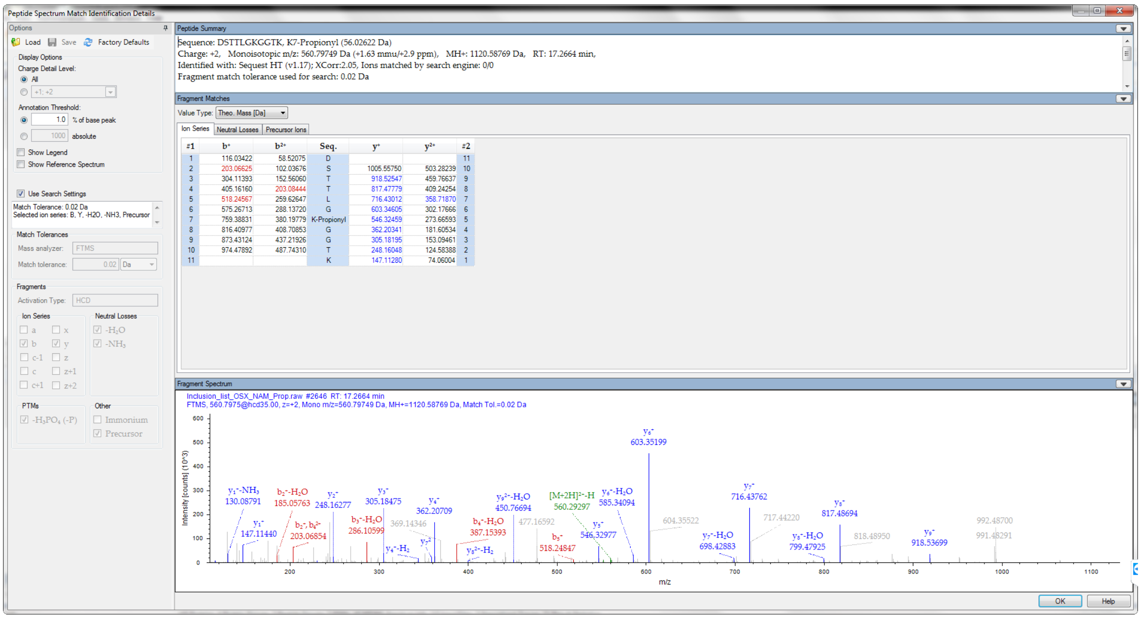

# K4Prop (OSX K45)

G G T k K P Y A D L S A P K

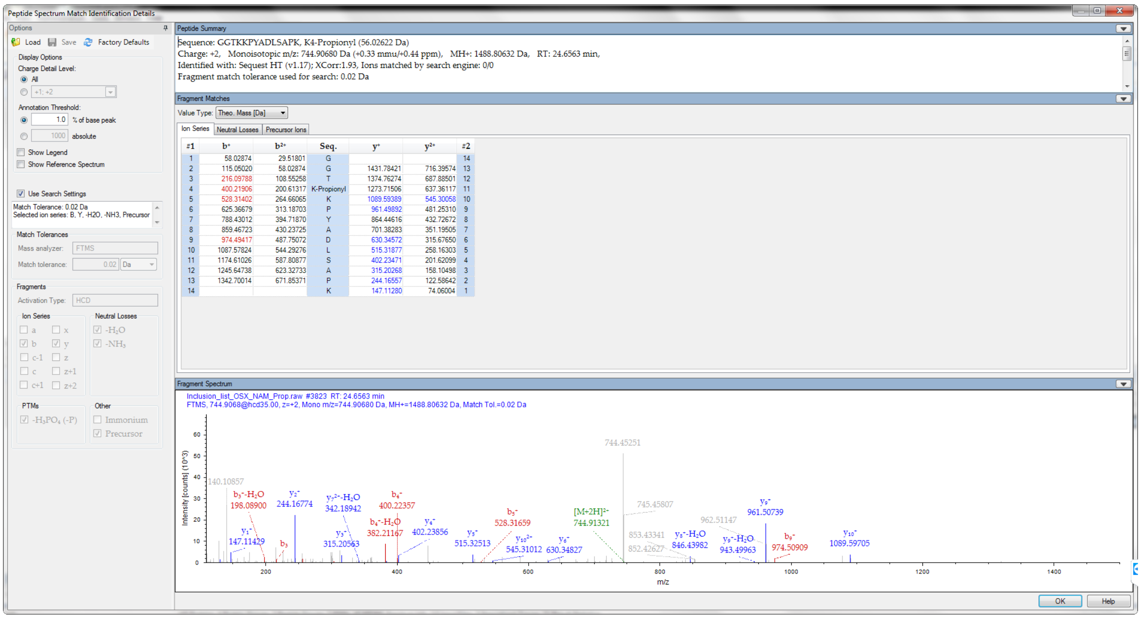


| #1 | b⁺ | b²⁺ | Seq. | y⁺ | y²⁺ | #2 |
|---|---|---|---|---|---|---|
| 1 | 58.02874 | 29.51801 | G | | | 14 |
| 2 | 115.05020 | 58.02874 | G | 1431.78421 | 716.39574 | 13 |
| 3 | 216.09788 | 108.55258 | T | 1374.76274 | 687.88501 | 12 |
| 4 | 400.21906 | 200.61317 | K-Propionyl | 1273.71506 | 637.36117 | 11 |
| 5 | 528.31402 | 264.66065 | K | 1089.59389 | 545.30058 | 10 |
| 6 | 625.36679 | 313.18703 | P | 961.49892 | 481.25310 | 9 |
| 7 | 788.43012 | 394.71870 | Y | 864.44616 | 432.72672 | 8 |
| 8 | 859.46723 | 430.23725 | A | 701.38283 | 351.19505 | 7 |
| 9 | 974.49417 | 487.75072 | D | 630.34572 | 315.67650 | 6 |
| 10 | 1087.57824 | 544.29276 | L | 515.31877 | 258.16303 | 5 |
| 11 | 1174.61026 | 587.80877 | S | 402.23471 | 201.62099 | 4 |
| 12 | 1245.64738 | 623.32733 | A | 315.20268 | 158.10498 | 3 |
| 13 | 1342.70014 | 671.85371 | P | 244.16557 | 122.58642 | 2 |
| 14 | | | K | 147.11280 | 74.06004 | 1 |

# K8Prop (OSX K358)

F T c L L c S k R

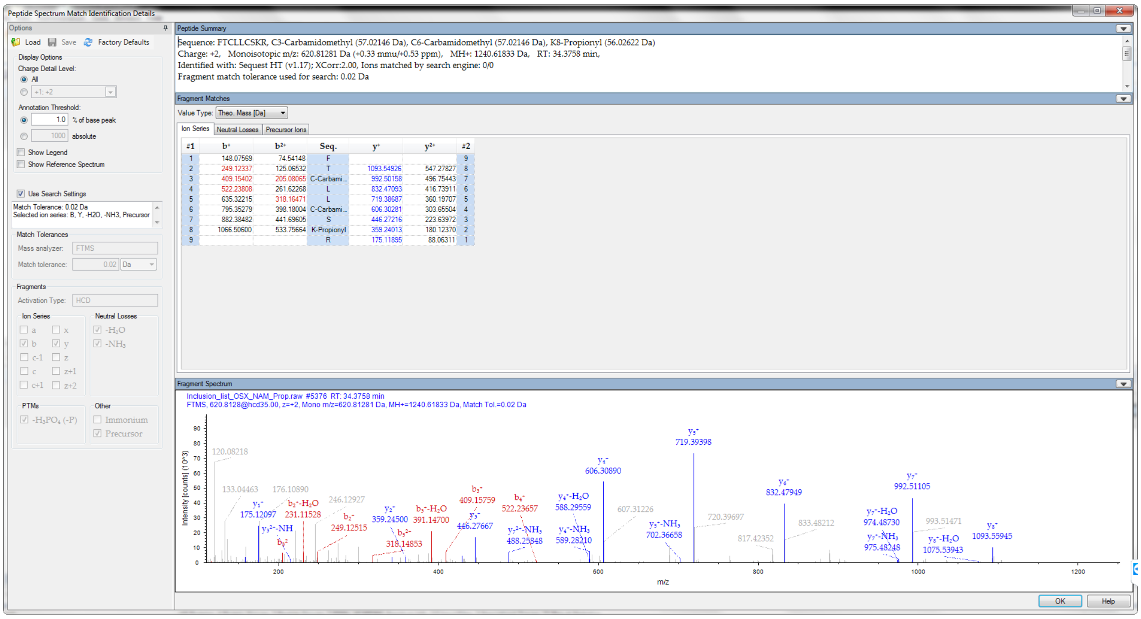


| #1 | b⁺ | b²⁺ | Seq. | y⁺ | y²⁺ | #2 |
|---|---|---|---|---|---|---|
| 1 | 148.07569 | 74.54148 | F | | | 9 |
| 2 | 249.12337 | 125.06532 | T | 1093.54926 | 547.27827 | 8 |
| 3 | 409.15402 | 205.08065 | C-Carbami... | 992.50158 | 496.75443 | 7 |
| 4 | 522.23808 | 261.62268 | L | 832.47093 | 416.73911 | 6 |
| 5 | 635.32215 | 318.16471 | L | 719.38687 | 360.19707 | 5 |
| 6 | 795.35279 | 398.18004 | C-Carbami... | 606.30281 | 303.65504 | 4 |
| 7 | 882.38482 | 441.69605 | S | 446.27216 | 223.63972 | 3 |
| 8 | 1066.50600 | 533.75664 | K-Propionyl | 359.24013 | 180.12370 | 2 |
| 9 | | | R | 175.11895 | 88.06311 | 1 |

**K15Prop (OSX K386)**

T H G E P G P G P P P S G P k E L G E G R

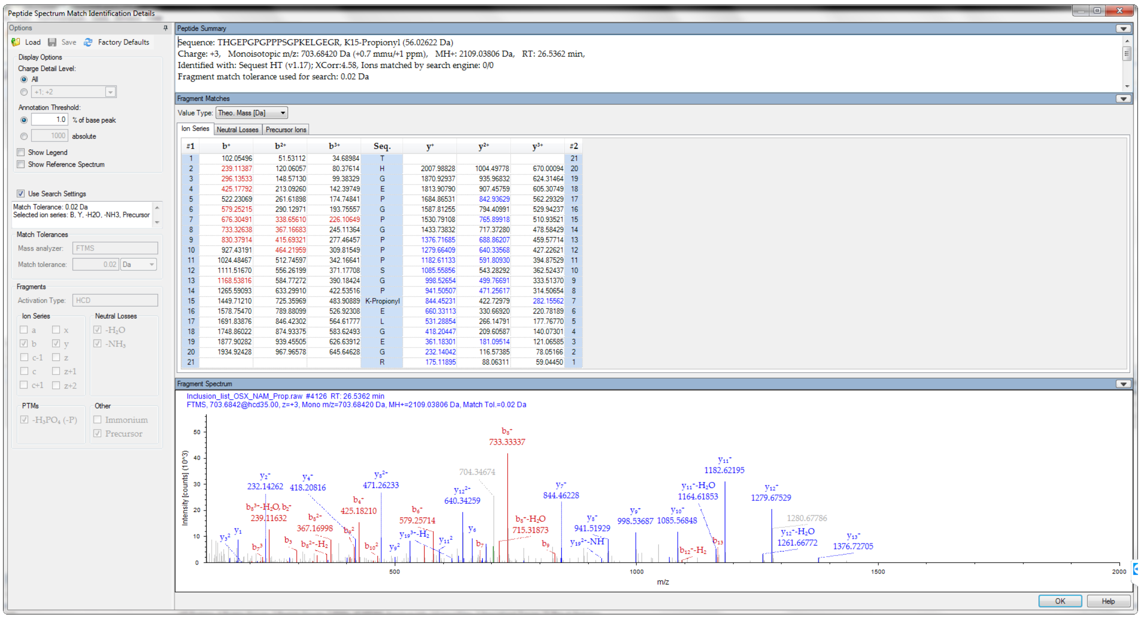


**Supplementary Figure 6: Identification of lysine propionylation in OSX.**

(**a**) Proteomic identification of propionylated and acetylated lysine residues in OSX. NanoLC-MS/MS analysis for the extracted peptides from the gel band corresponding OSX separated by SDS-PAGE was performed by Orbitrap (MS)-Iontrap (MS/MS) data dependent analysis mode using Uniprot mouse database. Identified peptides are shown with a green background. The total coverage was 48.83%. The peptide false discovery rate was less than 1%. A: Acetyl (K), C: Carbamidomethyl (C), O: Ixidation (M), P: propionyl (K)

(**b**) MS/MS spectra of propionyl-peptides in OSX identified by Orbitrap (MS)-Orbitrap (MS/MS) target mode analysis using inclusion list obtained from the Orbitrap (MS)-Iontrap (MS/MS) data dependent analysis. Four propionyl-peptides (m/z=560.7973, 620.8126, 703.6846, 744.908) were confirmed by the target mode analysis with high confidence (XCorr > 1.9). Inclusion list used were as follows, m/z=529.8029 VYG$^{304}$K$^{Prop}$ASHLK, 560.7973 DSTTLG$^{41}$K$^{Prop}$GGTK, 572.9647 $^{287}$K$^{Prop288}$K$^{Ac}$PIHSC$^{Cam}$HIPGC$^{Cam}$GK / $^{287}$K$^{Ac288}$K$^{Prop}$PIHSC$^{Cam}$HIPGC$^{Cam}$GK, 620.8126 FTC$^{Cam}$LLC$^{Cam}$S$^{358}$K$^{Prop}$R, 703.6846 THGEPGPGPPPSGP$^{386}$K$^{Prop}$ELGEGR, 744.908 GGT$^{45}$K$^{Prop}$KPYADLSAPK).

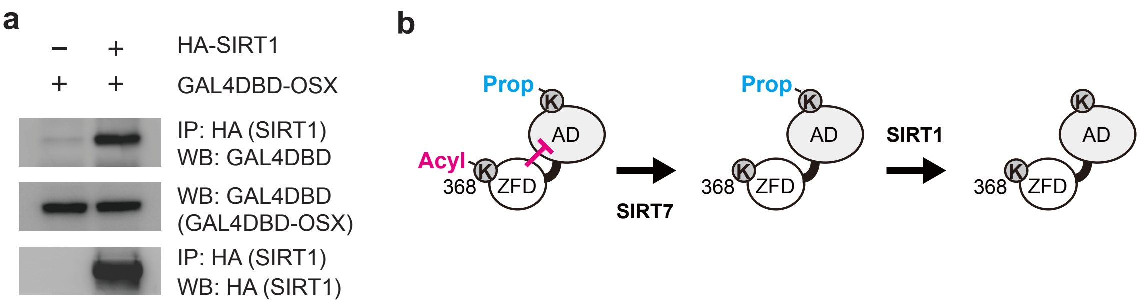


**Supplementary Figure 7: SIRT7 and SIRT1 regulate OSX transactivation activity through lysine deacylation.**

(**a**) Co-immunoprecipitation assay to assess the interaction between HA-SIRT1 and GAL4DBD-OSX in HEK293T cells.

(**b**) Proposed model for regulation of the transactivation activity of OSX by SIRT7 and SIRT1 through lysine deacylation.

WB=western blotting, IP=immunoprecipitation.

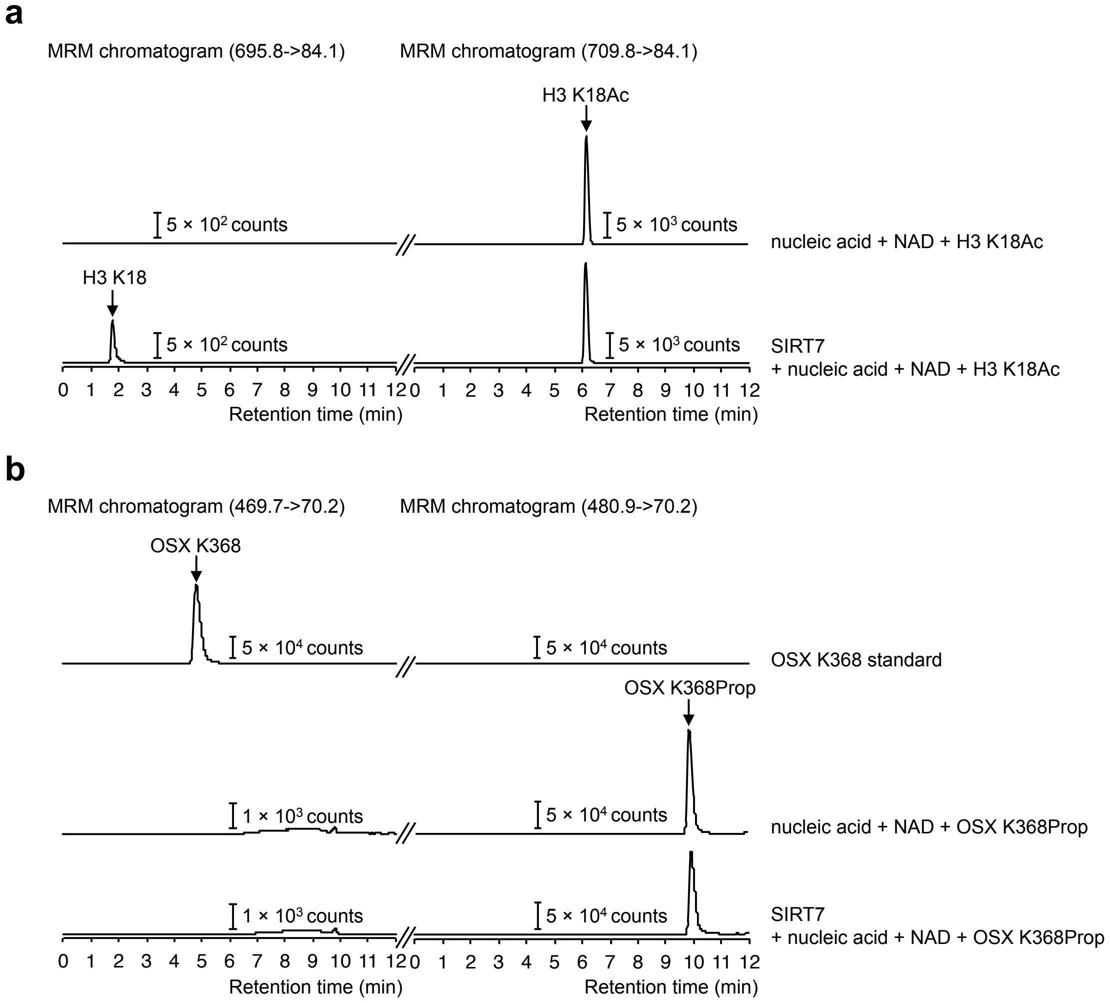


**Supplementary Figure 8: LC-MS/MS analysis of H3 K18 and OSX K368 peptides**

(**a**) Multiple reaction monitoring (MRM) chromatogram with m/z 695.8->84.1 (H3 K18) (left) and with m/z 709.8->84.1 (H3 K18Ac) (right), of the reaction mixture of nucleic acid, $NAD^+$ and H3 K18Ac with or without SIRT7.

(**b**) MRM chromatogram with m/z 469.7->70.2 (OSX K368) (left) and with m/z 480.9->70.2 (OSX K368Prop) (right), of OSX K368 standard and the reaction mixture of nucleic acid, $NAD^+$ and OSX K368Prop with or without SIRT7.

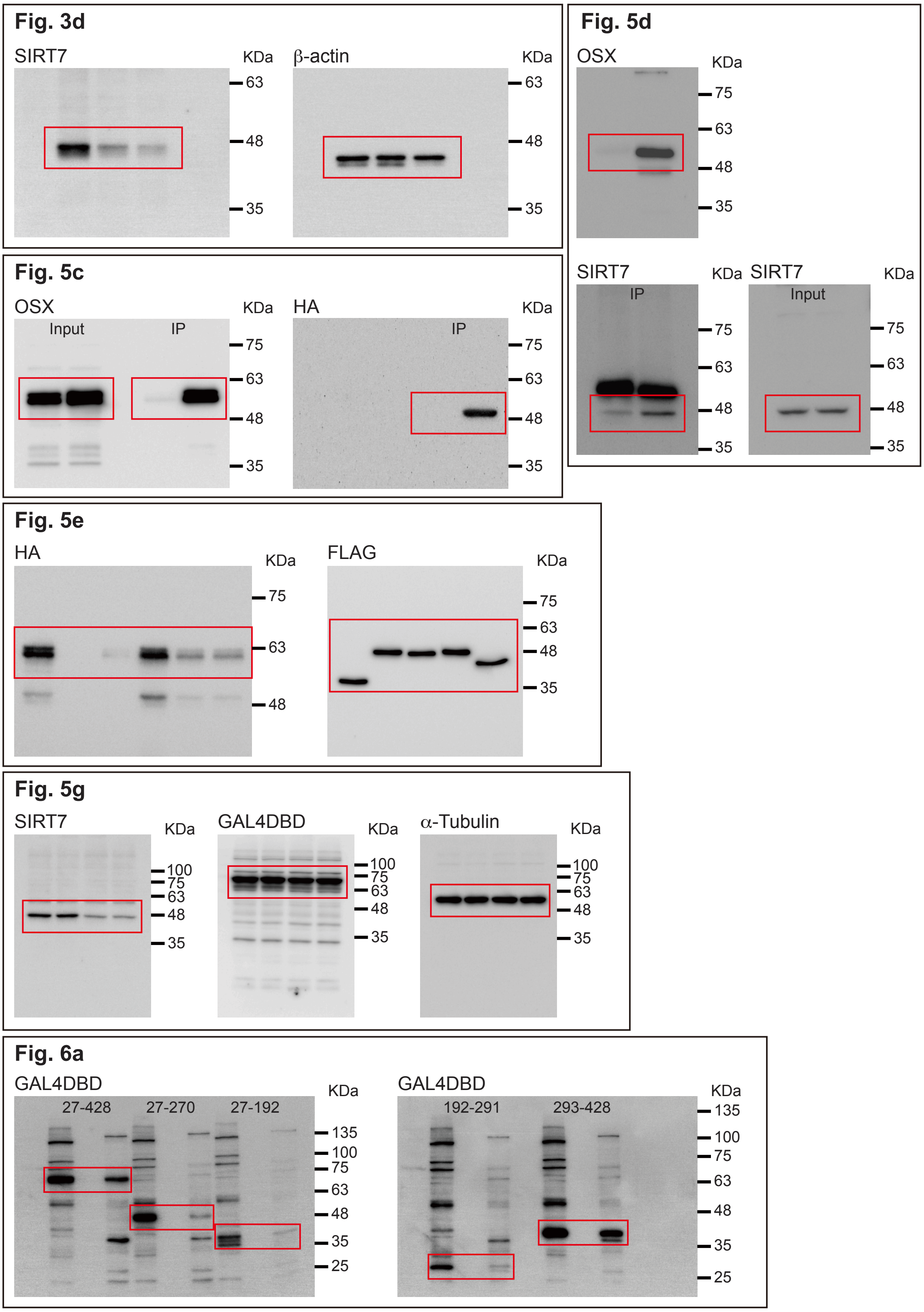
Fig. 3d
SIRT7
KDa
63
48
35
β-actin
KDa
63
48
35
Fig. 5d
OSX
KDa
75
63
48
35
SIRT7
IP
KDa
75
63
48
35
SIRT7
Input
KDa
75
63
48
35
Fig. 5c
OSX
Input
IP
KDa
75
63
48
35
HA
IP
KDa
75
63
48
35
Fig. 5e
HA
KDa
75
63
48
FLAG
KDa
75
63
48
35
Fig. 5g
SIRT7
KDa
100
75
63
48
35
GAL4DBD
KDa
100
75
63
48
35
α-Tubulin
KDa
100
75
63
48
35
Fig. 6a
GAL4DBD
27-428
27-270
27-192
KDa
135
100
75
63
48
35
25
GAL4DBD
192-291
293-428
KDa
135
100
75
63
48
35
25

Fig. 7a

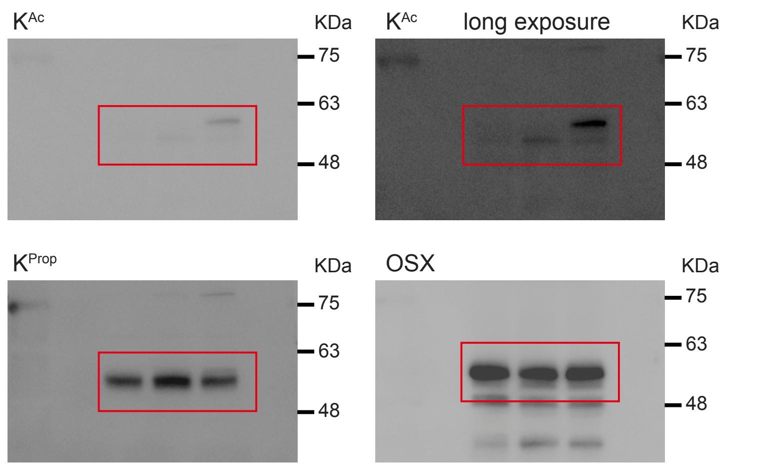


Fig. 7b

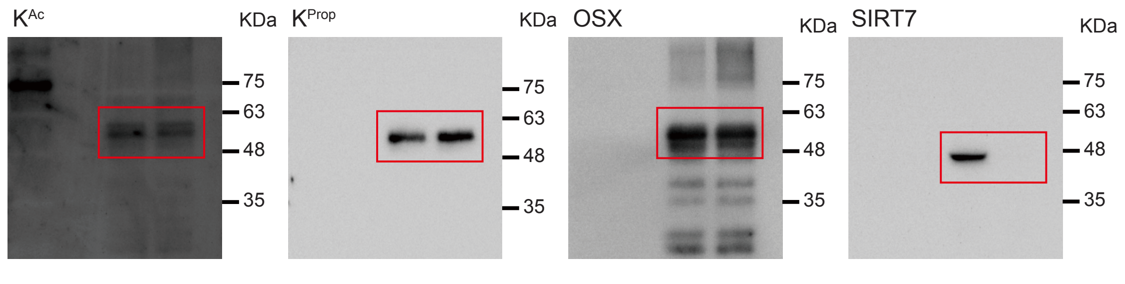


Fig. 7c

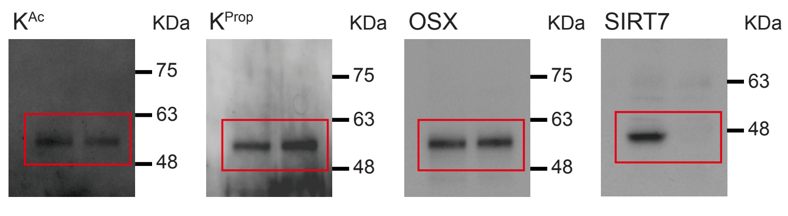


Fig. 7d

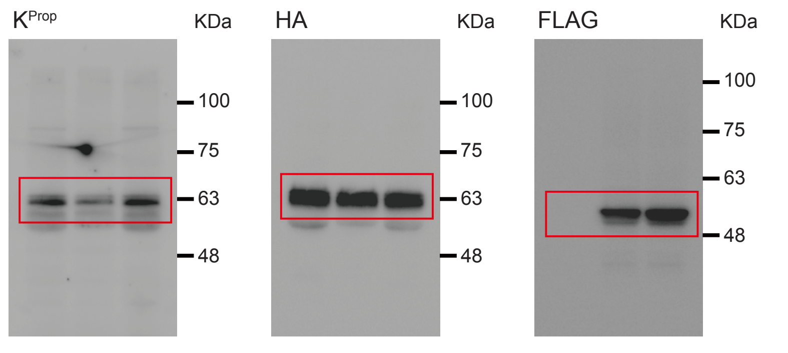


Fig. 7e

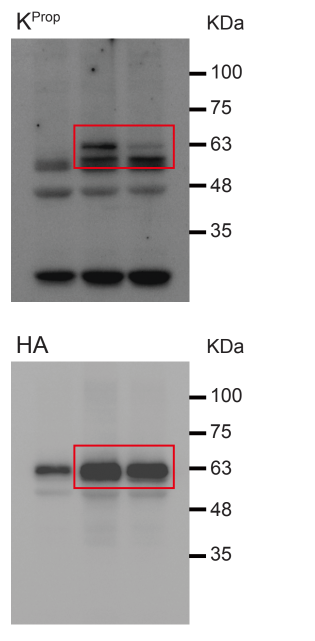

Fig. 7f

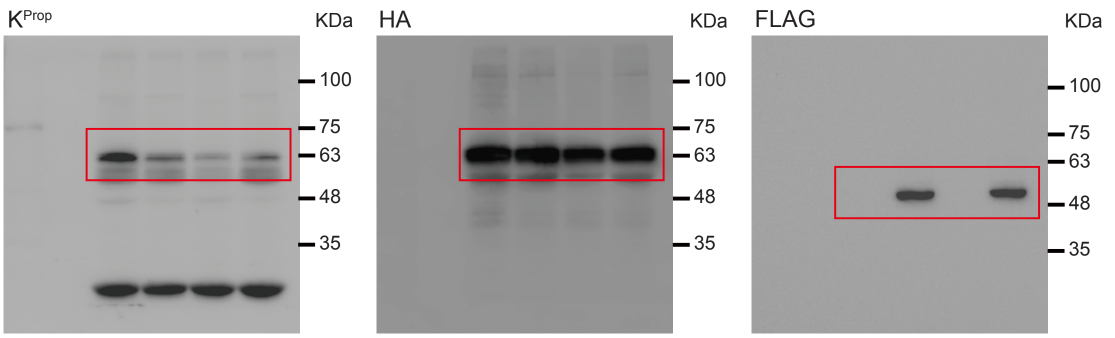


Fig. 7h

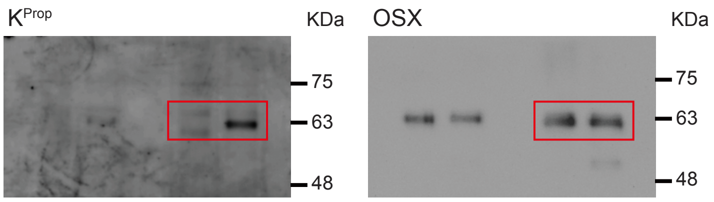


Fig. 7i

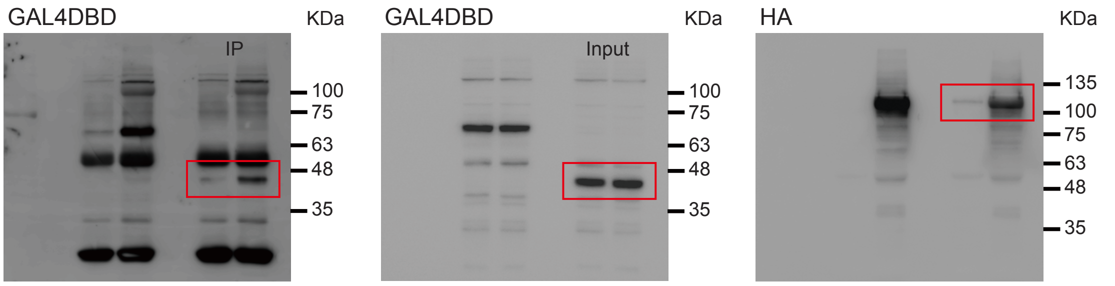


Fig. 7j

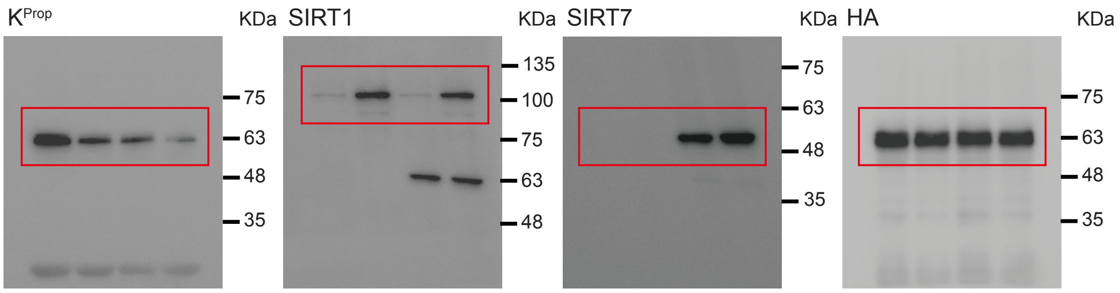

## Supplementary Fig. 3c

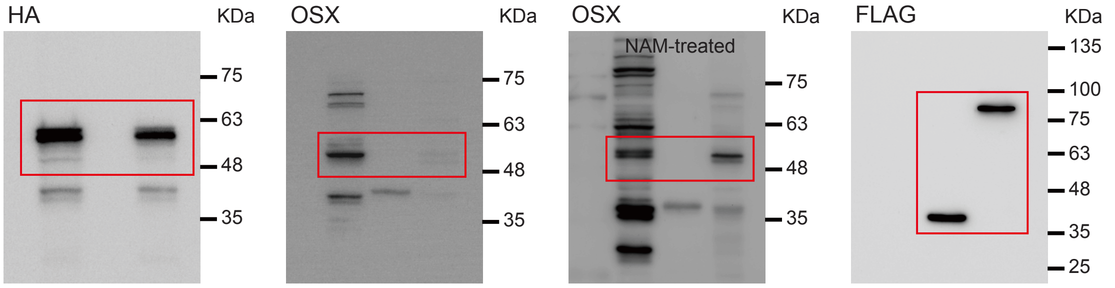


## Supplementary Fig. 4a

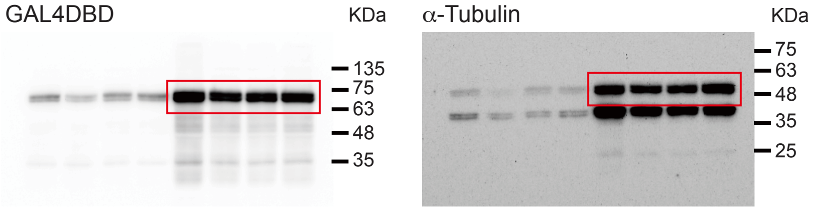


## Supplementary Fig. 5a

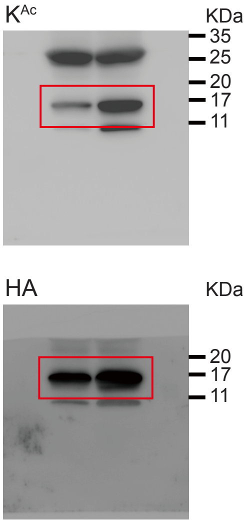


## Supplementary Fig. 5b

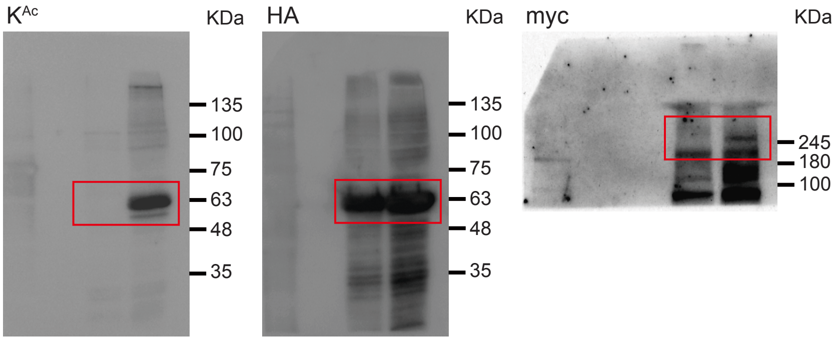


## Supplementary Fig. 5c

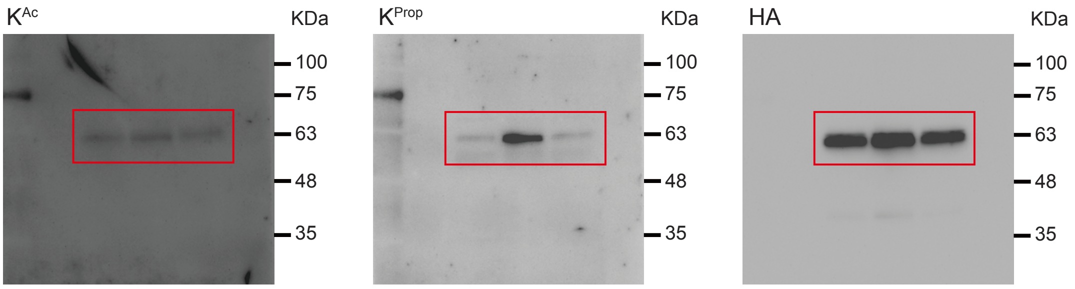

Supplementary Fig. 5d

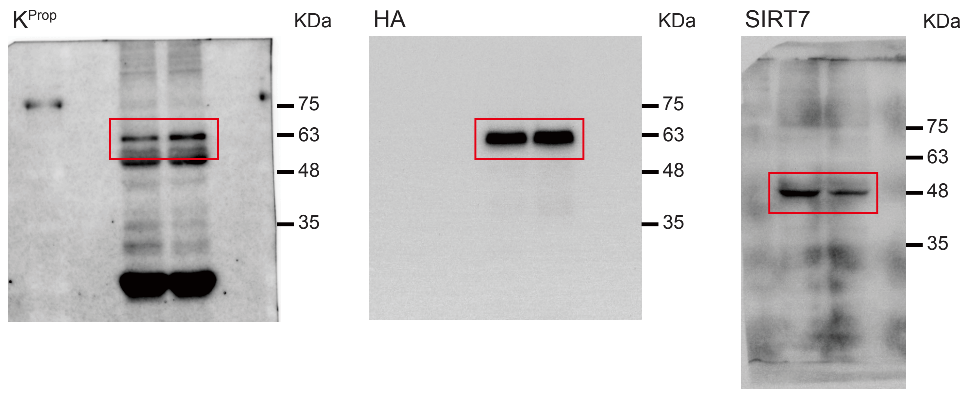


Supplementary Fig. 5e

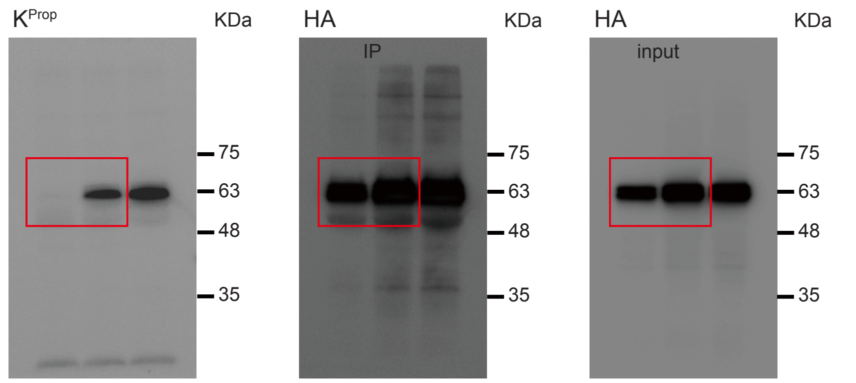


Supplementary Fig. 7a

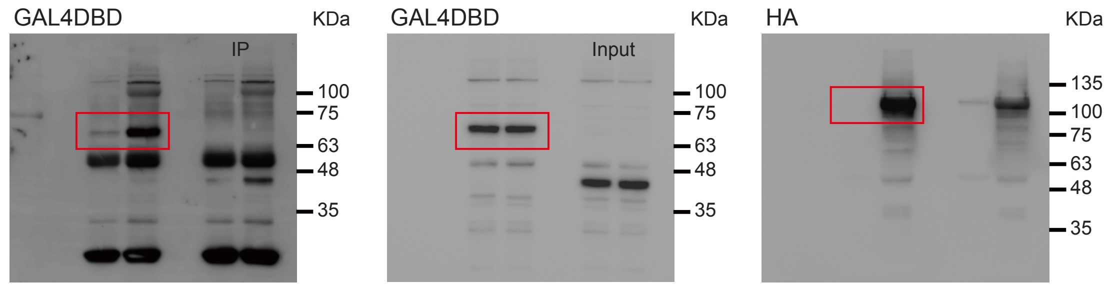


**Supplementary Figure 9: Full scans of all immunoblots**

Red boxes indicate the cropped region of the corresponding figures.

# Supplementary Table 1: Primer sequences used in qRT-PCR.

| Symbol | Name | Sequence | |
|---|---|---|---|
| | | Forward | Reverse |
| *Tbp* | TATA box binding protein | ACCCTTCACCAATGACTCCTATG | TGACTGCAGCAAATCGCTTGG |
| *Sirt1* | sirtuin 1 | GCATAGATACCGTCTCTTGATCTGAA | TGTGAAGTTACTGCAGGAGTGTAAA |
| *Sirt6* | sirtuin 6 | GGGACCTGATGCTCGCTGAT | CAGAGGTGGCAGGGCTTTGT |
| *Sirt7* | sirtuin 7 | TGCCAGGCACTTGGTTGTCT | TAGGCTCCGCTTCGCTTAGG |
| *Alp* | alkaline phosphatase, liver/bone/kidney | TAACACCAACGCTCAGGTCC | TGGATGTGACCTCATTGCCC |
| *Bsp* | integrin binding sialoprotein | AGGTGCAGAAGGAACCACAG | TCTCCCCCATACTCAACGGT |
| *Col1a1* | collagen, type I, alpha 1 | GGAGAGAGCATGACCGATGG | CGATCTCGTTGGATCCCTGG |
| *Ocn* | osteocalcin | CTGCGCTCTGTCTCTCTGAC | CTCTGGCCACTTACCCAAGG |
| *Osx* | Osterix | TTCGCATCTGAAAGCCCACT | TGCGCTGATGTTTGCTCAAG |
| *Runx2* | Runt-related transcription factor 2 | GCCACCTTTACCTACACCCC | ACTCTGGCTTTGGGAAGAGC |
| *Ctsk* | cathepsin K | TACCCATATGTGGGCCAGGA | TTCAGGGCTTTCTCGTTCCC |
| *Tcirg1* | T cell, immune regulator 1, ATPase, H+ transporting, lysosomal V0 protein A3 | GGGAAGTTAACCCTGCTCCC | GAAGCTTGTCCGTGTCCTCA |
| *Rankl* | tumor necrosis factor (ligand) superfamily, member 11 | GAAACTCACAGCCCTCTCTCTTG | GCATCGGAATACCTCTCCCAATC |
| *Opg* | tumor necrosis factor receptor superfamily, member 11b (osteoprotegerin) | AGGAACTGCAGTCCGTGAAG | ATTCCACACTTTTGCGTGGC |
| *B2m* | beta-2 microglobulin | TTCTGGTGCTTGTCTCACTG | TATGTTCGGCTTCCCATTCT |
| *Actb* | actin, beta | GGCCGGGACCTGACAGACTA | AGGAAGAGGATGCGGCAGTG |
| *18S* | 18S ribosomal RNA | GGAGAACTCACGGAGGACGA | CCAGTGGTCTTGGTGTGCTG |
